# Intraventricular hemorrhage prediction in premature neonates in the era of hemodynamics monitoring: a prospective cohort study

**DOI:** 10.1007/s00431-022-04630-5

**Published:** 2022-09-28

**Authors:** Marwa Mohamed Farag, Mohamed Hazem Gouda, Ali Mohamed Abd Almohsen, Mohammed Attia Khalifa

**Affiliations:** Pediatric Department, Faculty of Medicine, Alexandria University, Alexandria, Egypt

**Keywords:** Functional echocardiography, Preterm infant, Intraventricular hemorrhage, Superior vena cava flow, ACA resistive index, Trans-cranial Doppler

## Abstract

Unstable hemodynamics and prematurity are the main players in intraventricular hemorrhage (IVH) development. Our objective was to study 8 the use of superior vena cava flow (SVCF), left ventricular output (LVO), and right ventricular output (RVO), and anterior cerebral artery (ACA) Doppler measures in prediction of IVH in the first week of life in preterm infant ≤ 32 weeks and birth weight ≤ 1500 g. This prospective cohort study was conducted in 55NICU of Alexandria University maternity hospital. Of 147 enrolled patients, 132 infants born ≤ 32 weeks GA and birth weight ≤ 1500 g were eligible for- the study. One hundred twenty-seven infants completed the study. Infants were scanned for ACA-RI using transfontanellar ultrasound, and SVCF, LVO, and RVO using functional echocardiography in the first 24 h after birth. Patients had another two scans on DOL3 and 7 to detect IVH development. Low SVCF and high ACA-RI significantly increased the risk of IVH using logistic regression models with OR, 3.16; 95%CI, 1.19–8.39; *P* = 0.02 and OR, 1.64; 95%Cl, 1.10–2.44; *P* = 0.02, respectively. Low SVCF and high ACA-RI significantly increased risk of catastrophic IVH *P* = 0.025 and 0.023, respectively. Combined use of low SVCF < 55 ml/kg/min and ACA-RI > 0.75 is predictor of IVH with sensitivity 40.8% and 82.1% specificity.

*Conclusions*: There are strong relations between both low SVCF and high ACA-RI, and IVH development in premature neonates ≤ 32 weeks and birth weight ≤ 1500 g, with more significance towards catastrophic IVH. Admission RSS and LVO are the strongest factors affecting SVCF. Maternal anemia, patent ductus arteriosus size (mm/kg), and capillary refill time were significantly associated with high ACA-RI. These findings help in more understanding of pathophysiological factors affecting central perfusion that might affect the longer term neurodeveopmental outcome.

*Trial registration*: This work was registered in clinical trial.gv no *NCT05050032*.

**Table:** 

**What is Known:** *•Whether SVCF and RI-ACA can predict IVH in preterm neonates is still debatable.*	
**What is New:** *•Low SVC flow and high ACA-RI significantly increased risk of IVH, confirming the role of hypoperfusion-reperfusion cycle in IVH development. The most striking result that combined metrics using the cut-off value of < 41 ml/kg/min for SVCF and > 0.85 for ACA-RI “in the first day of life” can correctly reject the presence of IVH in 98% of patients “during the first week of life.”*

**Supplementary Information:**

The online version contains supplementary material available at 10.1007/s00431-022-04630-5.

## Introduction

Prediction and prevention of intraventricular hemorrhage (IVH) continue to be challenging in the care of preterm infants [1]. IVH is associated with high neonatal mortality and leads to severe long-term neurological sequelae. In developed countries, there is overall decreased incidence of IVH in preterm infants in the previous 5 decades due to the advances in neonatal intensive care [2], while in developing countries, data are scarce [3] and higher gestational ages and birth weight are included in the risk for IVH.

The absence of cerebral blood flow (CBF) autoregulation and hypoperfusion–reperfusion injury caused by systemic hemodynamic disturbances has been implicated in the pathogenesis of IVH in premature infants [4].

Functional echocardiography (FE) assessment of the cardiovascular function and systemic perfusion has been established as a monitoring tool for management of extremely preterm infants [5]. Transracranial Doppler (TCD) ultrasound can be used for assessment of cerebral blood flow and cerebral auto-regulation [6].

Those bedside diagnostic tools do not expose critically ill preterm infants to radiation, sedation, or transportation and might have a predictive and prognostic utility in IVH and immaturity-related brain injuries.

## Aim

The purpose of conducting the research was to evaluate the use of DOL1 superior vena cava flow (SVCF), left ventricular output (LVO), right ventricular output (RVO), and anterior cerebral artery (ACA) Doppler measures in prediction of IVH in the first week of life in preterm infant ≤ 32 weeks and birth weight ≤ 1500 g.

## Method

Preterm newborns with gestational age ≤ 32 weeks and birth weight ≤ 1500 g that were born in the university maternity hospital and admitted to the NICU were enrolled in this study in their first 24 h of life. Patients with congenital heart diseases and other major anomalies were excluded.

On DOL1, the recruited preterm infants had FE to assess SVCF, LVO, RVO, and ACA flow velocities through TCD. The studied infants were evaluated as regard the occurrence of IVH during the first week of life. Infants were categorized into two main groups: IVH group (*n* = 71) and no IVH group (*n* = 56).

The following clinical and laboratory data were obtained: perinatal history, ventilatory support (mode and MAP) and surfactant administration, scoring of severity of respiratory distress by Silverman Anderson’s respiratory severity score (RSS) at the time of admission, complete blood count (CBC), C-reactive protein (CRP), arterial blood gases, blood culture, prothrombin time (PT), and partial thromboplastin time (PTT).

At the time of the scan, all infants were assessed as regard heart rate, respiratory rate, peripheral pulsations, inotropic support, and capillary refill time (CRT) and assessed in three locations (forehead, mid−sternum, and big toe). Mild pressure was applied for 5 s; then, duration for color return was recorded. Non−invasive BP measurements (SBP, DBP, and MABP) using soft pressure cuffs were recorded.

All ultrasound studies were performed by a single examiner, who has 2-year training in neonatal echocardiography and cranial ultrasound, under the supervision of a consultant cardiologist and a consultant neonatologist, both experienced in neonatal functional echocardiography. All data were revised and approved by the two consultants. Approvals of medical ethical committee of Alexandria University Faculty of Medicine and parent medical ethical consent were obtained.

FE was performed on day 1 using 5–11 MHz GE, 12S-RS probe, GE Vivid iq premium machine with M, 2D, color Doppler modes, and pulsed wave (PW). The following parameters were measured:

1. SVCF:
2. Doppler volumetric measurements of the SVCF were measured by the methods of Kluckow and Evans [7]. A mean of the maximum and minimum diameter measured from a frozen two-dimensional (2D) image and averaged over 5 heart cycles. The velocity time integral (VTI) was calculated from the Doppler velocity tracings and averaged over 5 consecutive cardiac cycles. The heart rate was measured from the peak-to-peak intervals of the Doppler velocity time signals.
3. Low SVCF was defined as < 41 ml/kg/min within first 24 h of life [8].
4. LVO and RVO were measured using the Evans and Kluckow methodology. Low RVO and LVO were defined as < 150 ml/kg/min within first 24 h of life [9]. The size of the patent ductus arteriosus (PDA) was assessed from the ductal view. The duct was measured at its narrowest point, before its entry into the main pulmonary artery. PDA size, when indexed to the patient’s weight, > 1.4 mm/kg, together with left atrial-to-aortic root LA/Ao ratio was considered as markers of hemodynamically significant PDA (HS- PDA) [10].

Cranial ultrasound was done using the 3.5–10 MHz, GE 8C-RS probe, Vivid iq premium on DOL1, 3, and 7, and IVH was classified according to Papile grading [11]. ACA flow velocity measurement by TCD ultrasonography in DOL1 was assessed by placing the transducer in the midsagittal plane via the anterior fontanelle. Pulsed wave Doppler was done to measure peak systolic velocity (Vs) and maximum end-diastolic velocity (Vd) of ACA. The ACA- resistance index (ACA-RI) was calculated by the following equation: $\mathrm{ACARI} \, = \frac{\left\{\text{Vs \_Vd }\right\}}{\text{Vs}}$. High ACA-RI was defined as RI > 0.85 within first 24 h of life [6].

Data were fed to the computer and analyzed using the IBM SPSS software package version 20.0. The Kolmogorov–Smirnov test was used to verify the normality of distribution. Qualitative data were described using number and percent. Quantitative data were described using range (minimum and maximum), mean, and standard deviation, median, and interquartile range (IQR). Significance of the obtained results was judged at the 5% level. Two backward stepwise regression models were built to determine factors predicting IVH development with use of SVCF and ACA-RI, separately, as explanatory variables. Since both SVCF and ACA-RI were collinear, they were not included in the same model. Potential confounders were retained in the base models if they were statistically significant (*P* < 0.05) in the univariate analysis and can affect the explanatory variables both clinically and statistically (changing the OR by ≥ 10%). In case of collinearity between ≥ two variables, only one variable was retained in the model. Collinearity was found between PT and PTT, NCPAP and conventional ventilation, and RSS at admission, RR and MAP, in both models. The studied infants were further categorized into another two groups regardless to IVH status: low SVCF group (*n* = 44) and normal SVCF group (*n* = 83), and high ACA-RI group (*n* = 29) and normal RI group (*n* = 98) to study factors affecting central perfusion and CBF. Backward stepwise regression models were used, with variables included in the base models if they were statistically significant (*P* < 0.05) in the univariate analysis. In SVCF model, collinearity was found between NCPAP and conventional ventilation; RSS at admission, RR, and mean airway pressure; CRT and PP; and ACA-RI and ACA end diastolic velocity. In ACA-RI model, collinearity was found between RSS at admission and mean airway pressure. To evaluate the differences in IVH occurrence (no IVH, catastrophic and non-catastrophic IVH) according to SVCF and ACA-RI, Kruskal Wallis test and post hoc Dunn’s multiple comparisons test for pairwise comparisons were used. Sensitivity, specificity, PPV, and NPPV were calculated to assess the potentiality of low SVCF, high ACA-RI, and both of them in predicting IVH. Finally, receiver operating characteristic (ROC) curves and the concordance (C) statistic were used to assess the abilities of clinical (DBP, PT, RSS) and imaging (SVCF and ACA-RI) factors to predict IVH.

## Results

During the period from May 2020 till February 2021, 147 preterm newborns with GA ≤ 32 weeks and birth weight ≤ 1500 g were enrolled in this study. Only 127 infants were eligible and completed the study (S-Fig. 1). The study population was scanned in the first 24 h with mean time of scan 12.09 ± 6.61 (S-Table 2) and divided into groups according to development of IVH in first seven days after birth.

Table 1 summarizes the univariate analysis of demographic, resuscitation, initial laboratory, respiratory, and hemodynamic parameters of the studied groups. The rate of IVH was found to be significantly increased with lower GA and birth weight in preterm infants. The mean birth weight and the mean GA of infants in the IVH group were 1000 ± 220 g and 29.31 ± 1.56 weeks, respectively. Concerning imaging-based hemodynamic parameters, ACA-RI was strongly higher and SVCF and Vd velocity were strongly lower with IVH group. In the current work, the reported median values of SVCF in IVH group and non-IVH group were 44 and 59 ml/kg/min, respectively.

**Table 1:** Univariate logistic regression analysis for the IVH and no IVH groups

	**IVH**					***P***
	**No (*n* = 56)**		**Yes (*n* = 71)**		**OR (95%CI)**	
	**No**	**%**	**No**	**%**		
**Sex**						
Male	23	41.1	38	53.5	1.65 (0.81–3.35)	0.16
Female	33	58.9	33	46.5	1.0	
**SGA**						
No	29	51.8	33	46.5	1.0	0.55
Yes	27	48.2	38	53.5	1.24 (0.61–2.50)	
**GA (weeks)**						
Mean ± SD	30.3 ± 1.4		29.3 ± 1.6		0.66 (0.52–0.85)	0.001*
**Weight (kg)**						
Mean ± SD	1.1 ± 0.2		1 ± 0.2		0.11 (0.02–0.57)	0.009*
**Maternal medical history**						
Positive for anemia	23	41.1	28	39.4	0.93 (0.93–1.91)	0.85
Positive for infection	11	19.6	19	26.8	1.50 (0.64–3.47)	0.35
Positive for DM	0	0	3	4.2	–	0.999
Positive for preeclampsia	9	16.1	7	9.9	0.57 (0.20–1.64)	0.30
Negative for all	20	35.7	21	29.6	1.32 (0.63–2.79)	0.46
Gestational hypertension	3	5.4	3	4.2	0.78 (0.15–4.02)	0.77
**Antenatal steroids**						
Complete course	19	33.9	17	23.9	0.56 (0.24–1.28)	0.17
Incomplete course	14	25	17	23.9	0.76 (0.31–1.82)	0.53
None	23	41.1	37	52.1	1.0	
**Resuscitation data**						
Initial steps	47	83.9	38	53.5	1.0	
PPV	6	10.7	19	26.8	3.92 (1.42–10.78)	0.008*
ETT	3	5.4	14	19.7	5.77 (1.55–21.57)	0.009*
**Mode of delivery**						
NVD	14	25	29	40.8	2.07 (0.96–4.47)	0.06
CS	42	75	42	59.2	1.0	
**Sentinel event**						
Accidental hemorrhage	9	16.1	21	29.6	2.19 (0.91–5.27)	0.08
Cord prolapse	2	3.6	1	1.4	0.39 (0.03–4.37)	0.44
PTLP	23	41.1	32	45.1	1.18 (0.58–2.39)	0.65
PROM	13	23.2	13	18.3	0.74 (0.31–1.76)	0.50
ROM (< 18)	6	10.7	7	9.9	0.91 (0.29–2.88)	0.88
Non reassuring fetal heart rate tracing	3	5.4	2	2.8	0.51 (0.08–3.18)	0.47
Antepartum fit	3	5.4	1	1.4	0.25 (0.03–2.50)	0.24
Doppler abnormality	2	3.6	1	1.4	0.39 (0.03–4.37)	0.44
Obstructed labor	2	3.6	1	1.4	0.39 (0.03–4.37)	0.44
**RSS at admission**						
Mean ± SD	3.5 ± 1		4.6 ± 1.3		2.13 (1.52–2.99)	<0.001*
**MAP**						
Mean ± SD	4.4 ± 2.3		6.3 ± 3		1.31 (1.13–1.53)	<0.001*
**Respiratory support at time of scan**						
Conventional ventilation	10 (17.9%)		40 (56.3%)		5.94 (2.59–13.60)	<0.001*
HFOV	0 (0%)		2 (2.8%)		−	0.999
NCPAP	36 (64.3%)		22 (31%)		0.25 (0.12–0.52)	<0.001*
Minimal support	10 (17.9%)		7 (9.9%)		0.50 (0.18–1.42)	0.19
**Surfactant administration**						
No	51	91.1	52	73.2	1.0	0.02*
Yes	5	8.9	19	26.8	3.73 (1.29–10.74)	
**Inotropes (at the time of the scan)**						
No	53	94.6	39	54.9	1.0	<0.001*
Yes	3	5.4	32	45.1	14.50 (4.14–50.78)	
**Hb (g/dl)**						
Mean ± SD	15.4 ± 2.5		15.5 ± 2.3		1.02 (0.88–1.18)	0.82
**WBC (×10**^3^**/ul)**						
Mean ± SD	13.7 ± 11.6		13.8 ± 9.3		1.0 (0.97–1.04)	0.93
**Platelets (×10**^**3**^**/ul)**						
Mean ± SD	213.8 ± 77.3		203.6 ± 70		1.0 (0.99–1.003)	0.44
**CRP (s)**						
Mean ± SD	7.7 ± 8.3		13.5 ± 25.1		1.02 (0.99–1.05)	0.13
**PT (s)**						
Mean ± SD	16 ± 3.6		19.9 ± 9.4		1.12 (1.03–1.23)	0.01*
**PTT (s)**						
Mean ± SD	58 ± 14.5		69.8 ± 24.9		1.03 (1.01–1.05)	0.003*
**Initial BC**						
Negative	52 (92.9%)		64 (90.1%)		1.0	
Positive	4 (7.1%)		7 (9.9%)		1.42 (0.40 –5.12)	0.59
**HR (beat/min)**						
Mean ± SD	145 ± 14.5		153.6 ± 22.2		1.02 (1.004–1.05)	0.02*
**RR (breath/min)**						
Mean ± SD	52.1 ± 7.4		60.9 ± 12.5		1.09 (1.04–1.13)	<0.001*
**CRT (s)**						
Mean ± SD	2.4 ± 0.6		3 ± 0.9		3.04 (1.75–5.29)	<0.001*
**PP**						
Felt well	53 (94.6%)		54 (76.1%)		1.0	0.009*
Weak	3 (5.4%)		17 (23.9%)		5.56 (1.54–20.10)	
**SBP (mmHg)**						
Mean ± SD	55.4 ± 9.7		52 ± 10.2		0.97 (0.93–1.002)	0.06
**DBP (mmHg)**						
Mean ± SD	27.8 ± 7.9		24.6 ± 7.2		0.94 (0.899–0.99)	0.02*
**MABP (mmHg)**						
Mean ± SD	37.6 ± 8.8		33.5 ± 8.8		0.95 (0.91–0.99)	0.01*
**Initial ABG**						
**PH**						
Mean ± SD	7.3 ± 0.1		7.3 ± 0.1		0.04 (0.001–3.20)	0.15
**HCO**_**3**_						
Mean ± SD	21.6 ± 4		20.1 ± 3.5		0.89 (0.81–0.99)	0.03*
**PCO**_**2**_						
Mean ± SD	41 ± 9.3		41.1 ± 9.7		1.001 (0.96–1.04)	0.98
**SVC flow**						
Low (< 41)	11	19.6	33	46.5	3.55 (1.58–7.97)	0.002*
Normal (≥ 41)	45	80.4	38	53.5	1.0	
Mean ± SD	63.9 ± 31.6		51.5 ± 30.7		0.987 (0.975–0.999)	0.03*
**LVO**						
Low (< 150)	14	25	23	32.4	1.44 (0.66–3.15)	0.36
Normal (≥ 150)	42	75	48	67.6	1.0	
Mean ± SD	197.8 ± 74.2		191.2 ± 78.1		0.999 (0.994–1.003)	0.63
**RVO**						
Low (< 150)	0	0.0	9	12.7	−	0.999
Normal (≥ 150)	56	100.0	62	87.3	1.0	
Mean ± SD	298.1 ± 97.8		301.7 ± 135.4		1.0 (0.997–1.003)	0.87
**LA/AO ratio**						
Mean ± SD	1.1 ± 0.3		1.1 ± 0.3		1.11 (0.32–3.90)	0.87
**PDA/weight**						
Mean ± SD	1.2 ± 0.9		1.3 ± 1.1		1.14 (0.80–1.64)	0.46
**PFO size**						
Mean ± SD	1.5 ± 0.7		1.4 ± 0.8		0.97 (0.59–1.59)	0.91
**ACA peak systolic velocity (cm/s)**						
Mean ± SD	20.2 ± 6.8		18.2 ± 7.8		0.96 (0.92–1.01)	0.14
**ACA End diastolic velocity (cm/sec)**						
Mean ± SD	5.4 ± 3		4.1 ± 3.5		0.88 (0.79–0.99)	0.04*
**ACA resistance index**						
Mean ± SD	0.7 ± 0.1		0.8 ± 0.1		1.58 (1.13–2.21)	0.007*
Normal (≤ 0.85)	50	89.3	48	67.6	1.0	0.006*
High (> 0.85)	6	10.7	23	32.4	3.99 (1.496–10.66)	

*SGA* small for gestational age, *GA* gestational age, *DM* diabetes mellitus, *PPV* positive pressure ventilation, *ETT* endotracheal tube, *NVD* normal vaginal delivery, *CS* C-section, *PTLP* premature labour pain, *PROM* premature rupture of membranes, *MAP* mean airway pressure, *NCPAP* nasal continous positive airway pressure, *HFOV* high frequency oscillatory ventillation, *MABP* mean arterial blood pressure, *SBP* systolic blood pressure, *DBP* diastolic blood pressure, *ABG* arterial blood gas, *BC* blood culture, *PP* peripheral pulses, *PTT(s)* partial thromboplastin time in seconds, *PT* prothrobin time

*Statistically significant at *p* ≤ 0.05

Based on backward stepwise regression analysis (Table 2a), SVCF, along with resuscitation needs, PPV, PT, and inotropic support at the time of scan, are the most significant independent factors associated with IVH, with OR (95% CI) of 3.16 (1.19–8.39), 4.16 (1.31–13.16), 1.13 (1.02–1.26), and 11.61 (3.07–43.87)., respectively. Another elimination regression model was constructed using ACA-RI as an explanatory variable (Table 2b) and showed that ACA-RI together with respiratory rate, PT, and inotropic support at the time of scan are significantly associated with IVH with odd ratios OR (95% CI) 1.64 (1.10–2.44), 1.05 (1.001–1.10), 1.11 (1.01–1.21), and 8.97 (2.34–34.38), respectively.

**Table 2:** Multivariate binary logistic regression analysis was performed to determine factors predicting IVH development. Backward stepwise regression was constructed, with potential confounders that were retained in the base models if they changed the effect of the explanatory variables (SVCF and ACA-RI) both clinically and statistically (changing the OR by ≥ 10%). All the included variables were statistically significant (*P* < 0.05) in the univariate analysis. In case of collinearity between two or more variables, only one variable was retained in the model. Collinearity was found between PT and PTT, NCPAP and conventional ventilation, and RSS at admission, RR, and mean airway pressure in both models. Panel a shows multivariate logistic regression analysis (final model) for the effect of low SVC flow on IVH development. Variables in the base model gestational age, resuscitation data, respiratory support (NCPAP), and inotropes at the time of scan, RR, PT, and low SVC flow. Panel b shows multivariate logistic regression analysis (final model) for the effect of ACA RI on IVH development. Variables in the base model were as follows: gestational age, resuscitation data, respiratory support (NCPAP), and inotropes at the time of scan, surfactant administration, RR, PT, initial HCO_3_, and ACA RI

	Multivariate	
	Adjusted OR (95% CI)	Adjusted *P*
**(a) Multivariate logistic regression analysis for the effect of low SVC flow on IVH development (final model)**		
**Resuscitation data**		
Initial steps	1.0	
PPV	4.16 (1.31–13.16)	0.02*
ETT	2.26 (0.50–10.25)	0.29
**Inotropes at time of scan**	11.61 (3.07–43.87)	<0.001*
**PT (s)**	1.13 (1.02–1.26)	0.02*
**SVCF(low)**	3.16 (1.19–8.39)	0.02*
**(b) Multivariate logistic regression analysis for the effect of ACA RI on IVH development (final model)**		
**RR**	1.05 (1.001–1.10)	0.04*
**Inotropes at time of scan**	8.97 (2.34–34.38)	0.001*
**PT (s)**	1.11 (1.01–1.21)	0.02*
**ACA RI**	1.64 (1.10–2.44)	0.02*

*PPV* positive pressure ventilation, *ETT* endotracheal tube, *PT(s)* prothrombin time in seconds

*Statistically significant at *p* ≤ 0.05

Patients were further subdivided, depending on the grade of IVH, into non-catastrophic IVH (grades I and II) and catastrophic IVH (grades III and IV). We found that SVCF was significantly lower and ACA RI was significantly higher in the catastrophic IVH group as compared to the no IVH group using Kruskal Wallis test (Table 3).

**Table 3:** Comparison between the 3 patient subgroups (no IVH, non-catastrophic IVH and catastrophic IV) as regard SVCF and ACA-RI using Kruskal Wallis test

	**IVH**		**No IVH (*n* = 56)**	***H***	***P***
	**Non catastrophic (*n* = 37)**	**Catastrophic (*n* = 34)**			
**SVC flow (ml/kg/min)**				7.401	0.025*
Min.–Max	13.0–117.0	5.0–165.0	9.0–174.0		
Mean ± SD	50.99 ± 21.73	52.12 ± 38.49	63.91 ± 31.60		
Median (IQR)	45.0 (35.0–65.0)	39.0 (29.0–70.0)	59.50 (46.50–72.50)		
**Significance between groups**	p1 (0.999), p2 (0.039*), p3 (0.141)				
**ACA-RI**				7.563	0.023*
Min.–Max	0.56–1.0	0.57–1.0	0.57–1.0		
Mean ± SD	0.78 ± 0.12	0.81 ± 0.13	0.74 ± 0.09		
Median (IQR)	0.77 (0.71–0.87)	0.79 (0.72–0.93)	0.74 (0.67–0.81)		
**Significance between groups**	p1 (0.999), p2 (0.024*), p3 (0.267)				

*H H* for Kruskal Wallis test, pairwise comparison between every 2 groups were done using Dunn-Bonferroni post hoc method, *p1 P*-value for comparing between catastrophic and non-catastrophic, *p2 P*-value for comparing between catastrophic and no IVH, *p3 P*-value for comparing between non-catastrophic and no IVH

^*^Statistically significant at *P* ≤ 0.05

A cut-off value of < 55 ml/kg/min for SVCF and > 0.75 for ACA-RI in DOL 1 were computed using ROC curve for the occurrence of IVH in premature infants. IVH can be correctly predicted in 66.2% of patients with low SVCF and 59.2% of patients with high ACA-RI. The highest specificity 98.2 and PPV 93.3 were reached in combined metrics using the cut-off value of < 41 ml/kg/min for SVCF and > 0.85 for ACA-RI. This is most striking result as it means the absence of low SVCF and high ACA-RI can correctly reject presence of IVH in 98% of patients (Table 4).

**Table 4:** The predictive values of SVCF and ACA RI for IVH development using different cut-off values, and receiver operating characteristic (ROC) curves are demonstrated in S-Fig. 14

	**AUC**	**95% CI**	**Cut-off**	**Sensitivity**	**Specificity**	**PPV**	**NPV**
**SVC flow**	0.638*	0.542–0.735	< 55	66.2	60.7	68.1	58.6
**ACA RI**	0.634*	0.609–0.790	> 0.75	59.2	60.7	65.6	54
**Combined RI and SVC flow**				40.8	82.1	74.4	52.3
**SVC flow**	0.638*	0.542–0.735	< 41	46.5	80.4	75	54.2
**ACA RI**	0.634*	0.609–0.790	> 0.85	32.4	89.3	79.3	51
**Combined RI and SVC flow**				19.7	98.2	93.3	49.1

*Statistically significant at *p* ≤ 0.05

Patients were stratified, regardless their IVH status, according to the definition of low SVCF, < 41 ml/kg/min and high ACA-RI > 0.85. Two elimination logistic regression models were constructed to study different parameters affecting SVCF (S-Tables 3 and 4) and ACA-RI (S-Tables 5 and 6), therefore, drawing attention to factors influencing central perfusion and cerebral blood flow. We found that the strongest parameters affecting SVCF were admission RSS and LVO with OR (95% CI) 0.45 (0.30–0.68) and 1.02 (1.01–1.024), respectively. Maternal anemia, DA size (mm/kg), and CRT were significantly associated with high ACA-RI.

S-Fig. 15A shows a significantly moderate linear correlation between SVCF and LVO (*r* = 0.427, *P* < 0.001). However, RVO is weakly correlated with SVCF (*r* = 0.189, *P* < 0.033). (S-Fig. 15B).

There was no additional predictive value of each imaging marker over the clinical markers separately in IVH prediction, as shown in S-Fig. 16 and S-Table 7.

## Discussion

We present a prospective observational study examining the capability of hemodynamic measures in first day of life in predicting risk of developing IVH in the first 7 days of life in preterm infants born 32 weeks’ GA or less and with birth weight ≤ 1500 g.

Cycle of ischemia followed by reperfusion is the hypothesis that can explain the increased risk of IVH in premature brain. Definition of hypotension and hypoperfusion in the preterm infant is a clinical conundrum. The use of the available clinical data such as measuring heart rate, CRT, and blood pressure in describing an infant’s hemodynamic alone might be erroneous. However, imaging-based measurement of cardiovascular function, together with continuous monitoring of clinical data, might be more accurate in diagnosis of compromised systemic blood flow (SBF), associated with the IVH development [11].

CBF of the preterm neonate with hypotension is more influenced by cardiac cycle changes than term neonates. Therefore, cardiac output (CO) is the main determinant of central perfusion [12]. Several studies tried to establish the relationship between CO measurements and IVH development. Noori et al. reported that patients in the IVH group had lower LVO during the first 12 h of monitoring which tended to normalize thereafter just before IVH development, *P* = 0.07 [13]. In contrast, Evans and Kluckow found that low RVO rather than LVO was significantly (*P* = 0.026) associated with grade 3 and 4 IVH [14]. In good agreement with their findings, Table 1 shows that only 9 of the 127 studied patients had low RVO (less than 150 ml/kg/min) and all of those 9 patients developed IVH. However, neither low RVO nor LVO were statistically significant with IVH occurrence with p values 0.87 and 0.63, respectively. This was in accordance with da Costa et al. [15].

Estimation of SBF using CO measurements is affected by the presence of DA and PFO in LVO and RVO, respectively. The superior vena cava provides a solution to the left-to-right shunting problem as it depicts blood flow to the upper body, including the brain, and is unaffected by the left-to-right shunting [7].

Many researchers tried to establish the relationship between SVCF in the preterm infants and development of IVH in the first days of life [7, 16, 17]. In this study, IVH patients’ mean SVCF was lower (51.5 ml/kg/min) compared with non-IVH patients’ (63.9 ml/kg/min) with *P* = 0.03. Among the 127 patients studied, 44 had low SVCF (41 ml/kg/min), 33 of whom developed IVH.A significant association was found between low SVCF and IVH occurrence with an OR of 3.16 (1.19–8.39), *P* = 0.02. This finding was previously reported by Kluckow and Evans, and Miletin and Dempsey [7, 16].

When compared to the group without IVH, the catastrophic IVH group (grades 3 and 4) had a significantly lower SVCF, *P* = 0.025 (Table 3). That was concordant with Kluckow and Evans who found that low SVCF was significantly associated with severe degree of IVH, *P* = 0.0001 [18].

S-Table 1 shows that IVH occurred early in 41 (58%) patients on day 1, 22 of them had normal SVCF. According to the hypoperfusion-reperfusion theory, those 22 patients might be in the reperfusion part of the cycle (SVCF has been already normalized just before the IVH event) and the hypoperfusion phase may have occurred previously, possibly during labor and delivery. The remaining 19 cases had low SVCF (still in hypoperfusion phase or in the transition between hypoperfusion and reperfusion) and IVH progressed to higher grades in 11 of them during the follow-up scans (the peak of reperfusion part of the cycle).

Some authors did not find strong association between low SVCF and IVH which might have some possible explanations. Holberton et al. [19], despite using alternative method of measuring SVCF and reporting a relatively high mean SVCF for the whole population(114 ml/kg/min), they used the same cut-off value 41 ml/kg/min for defining low SVCF. Only 6 out of the 161 studied patients were found to have low SVCF and 4 of them developed severe IVH (grade 3 or 4). Bates et al. [20] could have a potential confounding factor of inter-observer variability in their retrospective study. Popat et al. [21] used a different definition for low SVCF (< 55 mL/kg/min).

CBF alternations or disruption can result in ischemia or hemorrhage. Authors tried to measure CBF in different methods. Greisen et al. reported that global CBF in premature non-distressed infants measured with ^133^Xenon was 20 ml/100 g/ml [22]. Kehrer et al. calculated CBF from cross sectional area of extracranial internal carotid artery (ICA) and angle-corrected time-averaged flow velocity of intracranial ICA and reported to be around 23 ml/min in preterm infants between 28 and 31 weeks’ gestational age at day 1 [23], while CBF measured by phase-contrast magnetic resonance angiography and corrected for body weight ranged between 9.8 and 94.0 mL/min/kg (median 28.5 mL/min/kg) [24]. In the current work, the reported median SVCF in non-IVH group is 59 ml/kg/min. This means that cerebral fraction of SVCF in the non-IVH group 48.3% (28.5/59), using the median of Wagenaar et al. This is different from the previously reported CBF fraction of SVCF, 80% [7]. There is no known report for actual CBF in premature patients with IVH, and it is expected to be dynamic according to hypoperfusion or reperfusion stage.

Resistive indexes and cerebral velocities of intracranial vessels are indicator of CBF in premature infants as the absolute values of CBF cannot be calculated because it is difficult to determine the diameter of the intracranial vessels [23].

In preterm newborns, intracranial hemorrhage develops due to cerebrovascular hemodynamic alterations and fluctuating pattern in CBF velocity. Initially, CBF diminishes, with a high ACA-RI and vasoconstriction leading to infarction of germinal matrix vessels. This is followed by a low ACA-RI and vasodilation with bleeding of germinal matrix vessels [25, 26]. Baik-Schneditz et al. reported that higher ACA-RI were associated with lower cerebral tissue oxygenation and ischemia in preterm infants [27]. In the current work, the backward stepwise regression model in Table 2b shows that high ACA-RI (> 0.85) is significantly associated with IVH with an OR 1.64 (95% CI (1.10–2.44)). ACA-RI value > 0.75 has 59.2% sensitivity in predicting IVH (Table 4). Furthermore, patients with catastrophic IVH have significantly higher mean value of ACA-RI 0.81 ± 0.13, *P* = 0.024, comparing no IVH group. Bates and Miletin et al. could not identify any association between ACA measurements and IVH [16, 20].

Low SVCF and ACA-RI are reflections of systemic insults rather than a disease state. In order to understand the pathophysiological role of both hemodynamic markers in IVH development, risk factors affecting SVCF and ACA-RI need to be studied. In the current study, we constructed univariate and multivariate backward step regression models to identify the effect of different variables on SVCF and ACA-RI, consequently, identifying factors affecting central perfusion and CBF.

Severe respiratory disease associated with high mean airway pressure may affect blood flow in preterm infants by reducing cardiac output or venous return to the heart. We found that high RSS at admission was significantly associated with low SVCF. Osborn et al. and Miletin et al. reported that low SVCF was significantly associated with higher mean airway pressure in the first day of life with *P* < 0.001, and *P* = 0.026, respectively [17, 28]. It is well known that severe sepsis and septic shock are associated with low SVCF [29]. In the current work, culture proven early onset sepsis is associated with low SVCF with OR 0.22 (95%CI 0.05–1.04), *P* = 0.06. LVO is also additional factor that can affect SVCF with *P* < 0.001. Maternal anemia, PDA size, and CRT are significantly associated with high ACA-RI as they can alter the CBF (S-Table-6).

Camfferman et al. in their systematic review found that high RI can indicate HS-PDA. An average RI of 0.61–0.81 was found in a small PDA, whereas the mean RI varied between 0.78 and 1.2 in a large PDA [30]. There is a high risk of anemia during pregnancy, especially in countries with low and middle incomes, and it might affect fetal and/or neonatal cerebral hemodynamics and arterial velocities [31]. CRT is a measure of hyoperfusion that is associated with ACA-RI, confirming the impact of systemic hypoperfusion on CBF.

Correlation between SVCF and LVO depends on duct patency and the presence of HS-PDA. On the other hand, the correlation between SVCF and RVO depends on presence of PFO. In our study, 37 patients had a closed DA at the time of the scan and the majority of patients 91/127 had non-hemodynamically significant PDA (mean = 1.23 mm). This might lead to the significant positive moderate correlation between SVCF and LVO (*r* = 0.427, *P* = < 0.001). Weaker correlation was found between SVCF and RVO due to presence of PFO in most of studied patients 115/127. Bischoff et al. reported strong correlation between SVCF measured by the modified method and both LVO (*r* = 0.63, *P* = 0.012) and RVO (*r* = 0.635, *P* = 0.011) only in patients without PDA [32]. Similarly, Miletin and Dempsey found an insignificant poor correlation between SVCF, and LVO (*r* = 0.17, *P* = 0.30) and RVO (*r* = 0.14, *P* = 0.42) as they reported the presence of a PDA in almost all patients of their study [16].

In neonates, the imaging modalities are attractive tools that help the clinicians in active management of patients. Nevertheless, the users can face many challenges. First, hemodynamics in critically ill category like premature infants are changeable by time. Second, there is no added predictive value of radiological over clinical assessments, as shown in our results. Third, ultrasound evaluation carries risk of subjectivity, though being probably less subjective than clinical assessment. This is why it might be important to use the indispensable clinical tools in conjunction with those imaging tools.

## Conclusions

High > 0.85ACA-RI and low SVCF < 41 ml/kg/min within the first day of life are associated with increased risk of IVH development with more relation to catastrophic type of IVH in prematurely born infants and having birth weight ≤ 1500 g. Moreover, the combined use of those hemodynamic measures can correctly exclude the presence of IVH. There is a direct positive correlation between SVCF and LVO. Both LVO and the degree of respiratory distress, based on the Silverman Anderson’s score (RSS) at the time of admission, are the most important independent factors affecting SVCF, while PDA size, maternal anemia, and CRT are strongly associated with high ACA-RI in preterm newborns. While each of the clinical and imaging markers alone has its limitations, when used together, they can enhance prediction of IVH.

### Limitations

We recognize several limitations. First, there is high incidence of IVH in preterm infants and very low birth weight neonates in the present study. However, it reflects a real heath problem in developing countries that needs extensive study. Second, SVCF increases over the first 48 h in preterm infants and is highly variable in first 24 h. The scan was preformed once in the first 24 h, and scan timing was between 1 and 23 h which is relatively wide range. Nevertheless, the timing of the scan was not statistically significant in patients with and without IVH. Third, follow-up for long-term neurodevelopmental abnormalities was not feasible. Fourth, no therapeutic decision was taken based on hemodynamic parameters values as our study was an observational study.

## Supplementary Information

Below is the link to the electronic supplementary material.

Supplementary file1 (DOCX 2220 kb)

## Abbreviations

ACA-RI: Anterior cerebral artery resistive index
BP: Blood pressure
CBF: Cerebral blood flow
CRT: Capillary refill time
CO: Cardiac output
CBC: Complete blood count
CRP: C-reactive protein
DOL: Day of life
DA: Ductus arteriosus
DBP: Diastolic blood pressure
FE: Functional echocardiography
IVH: Intraventricular hemorrhage
L /A ratio: Left atrium to aortic root ratio
LVO: Left ventricular output
MABP: Mean arterial blood pressure
MAP: Mean airway pressure
PT: Prothrombin time
PTT: Partial thromboplastin time
PDA: Patent ductus arteriosus
PFO: Patent foramen ovale
RSS: Silverman Anderson’s respiratory severity score
RVO: Right ventricular output
SBF: Systemic blood flow
SBP: Systolic blood pressure
SVC flow: Superior vena cava flow
TCD: Transcranial Doppler
VTI: Velocity time integral
