# Supplementary material for: Intraventricular hemorrhage prediction in premature neonates in the era of hemodynamics monitoring: a prospective cohort study
Source: Eur J Pediatr. 2022 Sep 28;181(12):4067–77. doi: 10.1007/s00431-022-04630-5 (PMC9649466; doi:10.1007/s00431-022-04630-5)
Supplement: Supplementary file 1 — Supplementary file1 (DOCX 2220 kb) [file 431_2022_4630_MOESM1_ESM.docx]

D3 CUS

D7 CUS

D1 FE, TCD, CUS

**S-Figure (1): Study flow chart**

A total of 147 preterm newborns with GA ≤32 weeks and birth weight less than or equal 1500 g were enrolled in the study. Seven of these babies had congenital heart diseases. Out of the remaining 140 patients, 8 were excluded as they had congenital malformations. This left 132 infants who were eligible for recruitment. However, another 5 were excluded as they died before the seventh day of postnatal age and did not develop IVH before their death, leaving .Only127 infants for the analysis. Of the 127 , seven cases died before day3 and 19 patients died before day7 , however , they weren’t excluded because they developed IVH before death. The studied infants were evaluated as regards the occurrence of germinal matrix-intraventricular hemorrhages (GMH-IVH) during the first week of life. Infants were categorized into two main groups: IVH group (n=71) and no IVH group (n=56). Moreover, the studied infants were categorized into two different groups: low SVC flow group (n=44) and normal SVC flow group (n=83) to test the effect of different variables on SVC flow on the first day of life.

VSD ventricular septal defect ,PS pulmonary stenosis ,RHH right hypopalstic heart ,CUS cranial ultrasound,PCKD polycystic kidney diseases, FE functional echocardiography, TCD transcranial Doppler.

**S-Table (1): Distribution of the patients with IVH as regards IVH grade and onset of occurrence .**

|  | | **No.** | **%** |
| --- | --- | --- | --- |
| IVH grade | Grade 1 | 20 | 28.2% |
|  | Grade 2 | 17 | 23.9% |
|  | Grade 3 | 21 | 29.6% |
|  | Grade 4 | 13 | 18.3% |
| IVH onset | Day 1 | 41 | 57.7% |
|  | Day 3 | 20 | 28.2% |
|  | Day 7 | 10 | 14.1% |

**S-Table (2): comparison of time of scan in patients with normal and low SVC flow.**

|  | **Total (n = 127)** | **SVC flow** | | **OR (95%C.I)** | **P value** |
| --- | --- | --- | --- | --- | --- |
|  |  | **Low (n = 44)** | **Normal (n = 83)** |  |  |
| **Time of the scan** |  |  |  |  |  |
| Min. – Max. | 1.0 – 23.0 | 1.0 – 23.0 | 1.0 – 23.0 | 0.965 (0.912 – 1.021) | 0.216 |
| Mean ± SD. | 12.09 ± 6.61 | 13.09 ± 6.12 | 11.57 ± 6.83 |  |  |
| Median (IQR) | 12.0 (6.0 – 18.0) | 13.0 (9.0 – 18.5.0) | 11.0 (5.0 – 17.0) |  |  |

**S-Table (3): Univariate logistic regression analysis for the low and normal SVC flow groups**

|  | **SVC flow** | | | | **Univariate** | |
| --- | --- | --- | --- | --- | --- | --- |
|  | **Low (n = 44)** | | **Normal (n = 83)** | | **OR (95%C.I)** | **P value** |
|  | **No.** | **%** | **No.** | **%** |  |  |
| **Sex** |  |  |  |  |  |  |
| Male | 22 | 50 | 39 | 47 | 0.89 (0.43 – 1.84) | 0.75 |
| Female | 22 | 50 | 44 | 53 | 1.0 |  |
| **Gestational age** |  | |  | |  |  |
| Mean ± SD. | 29.3 ± 1.2 | | 30 ± 1.7 | | 1.34 (1.05 – 1.71) | 0.02^*^ |
| **Weight (kg)** |  | |  | |  |  |
| Mean ± SD. | 1 ± 0.2 | | 1.1 ± 0.2 | | 2.64 (0.50 – 13.92) | 0.25 |
| **Maternal medical history** |  |  |  |  |  |  |
| Anemia | 18 | 40.9 | 33 | 39.8 | 0.95 (0.45 – 2.01) | 0.90 |
| Infection | 13 | 29.5 | 17 | 20.5 | 0.61 (0.27 – 1.42) | 0.26 |
| DM | 1 | 2.3 | 2 | 2.4 | 1.06 (0.09 – 12.05) | 0.96 |
| Preeclampsia | 5 | 11.4 | 11 | 13.3 | 1.19 (0.39 – 3.68) | 0.76 |
| GHT | 1 | 2.3 | 5 | 6 | 2.76 (0.31 – 24.36) | 0.36 |
| Negative for all | 12 | 27.3 | 29 | 34.9 | 1.43 (0.64 – 3.20) | 0.38 |
| **Antenatal steroids** |  |  |  |  |  |  |
| Complete course | 12 | 27.3 | 24 | 28.9 | 1.43 (0.60 – 3.38) | 0.42 |
| Incomplete course | 7 | 15.9 | 24 | 28.9 | 2.45 (0.91 – 6.57) | 0.08 |
| None | 25 | 56.8 | 35 | 42.2 | 1.0 |  |
| **Resuscitation data** |  |  |  |  |  |  |
| Initial steps | 24 | 54.5 | 61 | 73.5 | 1.0 |  |
| PPVe | 9 | 20.5 | 16 | 19.3 | 0.70 (0.27 – 1.80) | 0.46 |
| ETT | 11 | 25 | 6 | 7.2 | 0.22 (0.07 – 0.65) | 0.006^*^ |
| **Mode of delivery** |  |  |  |  |  |  |
| NVD | 19 | 43.2 | 24 | 28.9 | 0.54 (0.25 – 1.15) | 0.11 |
| CS | 25 | 56.8 | 59 | 71.1 | 1.0 |  |
| **Sentinel event** |  |  |  |  |  |  |
| Accidental HGE | 12 | 27.3 | 18 | 21.7 | 0.74 (0.32 – 1.72) | 0.48 |
| Cord prolapse | 0 | 0 | 3 | 3.6 | – | 0.999 |
| PTLP | 17 | 38.6 | 38 | 45.8 | 1.34 (0.64 – 2.82) | 0.44 |
| PROM | 10 | 22.7 | 16 | 19.3 | 0.81 (0.33 – 1.98) | 0.65 |
| ROM (<18) | 6 | 13.6 | 7 | 8.4 | 0.58 (0.18 – 1.86) | 0.36 |
| Non reassuring fetal heart rate tracing | 1 | 2.3 | 4 | 4.8 | 2.18 (0.24 – 20.09) | 0.49 |
| Antepartum fit | 1 | 2.3 | 3 | 3.6 | 1.61 (0.16 – 15.98) | 0.68 |
| Doppler abnormality | 0 | 0 | 3 | 3.6 | – | 0.999 |
| Obstructed labor | 1 | 2.3 | 2 | 2.4 | 1.06 (0.09 – 12.05) | 0.96 |
| **RSS** **at admission** |  | |  | |  |  |
| Mean ± SD. | 4.8 ± 1.2 | | 3.8 ± 1.2 | | 0.49 (0.34 – 0.69) | <0.001^*^ |
| **MAP** |  | |  | |  |  |
| Mean ± SD. | 6.5 ± 3.1 | | 4.9 ± 2.6 | | 0.80 (0.69 – 0.93) | 0.004^*^ |
| **Respiratory support** |  | |  | |  |  |
| Conventional vent | 25 (56.8%) | | 25 (30.1%) | | 0.33 (0.15 – 0.70) | 0.004^*^ |
| HFOV | 2 (4.5%) | | 0 (0%) | | – | 0.999 |
| NCPAP | 13 (29.5%) | | 45 (54.2%) | | 2.82 (1.30 – 6.15) | 0.009^*^ |
| Minimal support^#^ | 4 (9.1%) | | 13 (15.7%) | | 1.86 (0.57 – 6.08) | 0.31 |
| **Hb (g/dl)** |  | |  | |  |  |
| Mean ± SD. | 15.9 ± 2.2 | | 15.2 ± 2.5 | | 0.87 (0.74 – 1.03) | 0.11 |
| **WBC (×10^3^/ul)** |  | |  | |  |  |
| Mean ± SD. | 12.8 ± 6.9 | | 14.3 ± 11.8 | | 1.02 (0.98 – 1.06) | 0.46 |
| **Platelets (×10^3^/ul)** |  | |  | |  |  |
| Mean ± SD. | 200 ± 80 | | 212.3 ± 69.4 | | 1.002 (0.997 – 1.01) | 0.37 |
| **CRP (sec.)** |  | |  | |  |  |
| Mean ± SD. | 13.8 ± 22.1 | | 9.4 ± 18.2 | | 0.99 (0.97 – 1.01) | 0.25 |
| **PT (sec)** |  | |  | |  |  |
| Mean ± SD. | 18.4 ± 8.6 | | 18.1 ± 7.1 | | 0.99 (0.95 – 1.04) | 0.80 |
| **PTT (sec)** |  | |  | |  |  |
| Mean ± SD. | 62.6 ± 20.5 | | 65.7 ± 22.4 | | 1.01 (0.99 – 1.03) | 0.45 |
| **Initial BC** |  | |  | |  |  |
| Negative | 37 (84.1%) | | 79 (95.2%) | | 1.0 |  |
| Positive | 7 (15.9%) | | 4 (4.8%) | | 0.27 (0.07 –0.97) | 0.045^*^ |
| **HR (beat/min)** |  | |  | |  |  |
| Mean ± SD. | 152.1 ± 22.7 | | 148.6 ± 17.8 | | 0.99 (0.97 – 1.01) | 0.34 |
| **RR (breath/min)** |  | |  | |  |  |
| Mean ± SD. | 60.8 ± 11.3 | | 55.1 ± 11 | | 0.96 (0.93 – 0.99) | 0.009^*^ |
| **CRT (sec)** |  | |  | |  |  |
| Mean ± SD. | 3.2 ± 0.9 | | 2.5 ± 0.7 | | 0.30 (0.18 – 0.52) | <0.001^*^ |
| **PP** |  | |  | |  |  |
| Felt well | 32 (72.7%) | | 75 (90.4%) | | 1.0 | 0.01^*^ |
| Weak | 12 (27.3%) | | 8 (9.6%) | | 0.28 (0.11 – 0.76) |  |
| **SBP (mmHg)** |  | |  | |  |  |
| Mean ± SD. | 55.1 ± 9.4 | | 52.7 ± 10.4 | | 0.98 (0.94 – 1.01) | 0.19 |
| **DBP (mmHg)** |  | |  | |  |  |
| Mean ± SD. | 27 ± 7.7 | | 25.5 ± 7.6 | | 0.98 (0.93 – 1.02) | 0.31 |
| **MABP (mmHg)** |  | |  | |  |  |
| Mean ± SD. | 36.2 ± 8.8 | | 34.8± 9.1 | | 0.98 (0.94 – 1.02) | 0.41 |
| **LVO (ml/kg/min)** |  |  |  |  |  |  |
| Low (<150) | 20 | 45.5 | 17 | 20.5 | 0.31 (0.14 – 0.69) | 0.004^*^ |
| Normal (≥150) | 24 | 54.5 | 66 | 79.5 | 1.0 |  |
| Mean ± SD. | 155.2 ± 56 | | 214.8 ± 77.6 | | 1.01 (1.01 – 1.02) | <0.001^*^ |
| **RVO (ml/kg/min)** |  |  |  |  |  |  |
| Low (<150) | 8 | 18.2 | 1 | 1.2 | 0.06 (0.01 – 0.46) | 0.007^*^ |
| Normal (≥150) | 36 | 81.8 | 82 | 98.8 | 1.0 |  |
| Mean ± SD. | 268.6 ± 135.2 | | 316.8 ± 108.1 | | 1.004 (1.001 – 1.01) | 0.03^*^ |
| **LA/AO ratio** |  | |  | |  |  |
| Mean ± SD. | 1.1 ± 0.3 | | 1.2 ± 0.3 | | 1.28 (0.34 – 4.82) | 0.72 |
| **PDA/weight (mm/kg)** |  | |  | |  |  |
| Mean ± SD. | 1.2 ± 1.1 | | 1.2 ± 0.9 | | 1.02 (0.71 – 1.48) | 0.91 |
| **PFO Size (mm)** |  | |  | |  |  |
| Mean ± SD. | 1.3 ± 0.7 | | 1.5 ± 0.7 | | 1.63 (0.96 – 2.76) | 0.07 |
| **Peak systolic velocity** |  | |  | |  |  |
| Mean ± SD. | 17.4 ± 7.7 | | 20 ± 7.2 | | 1.06 (0.997 – 1.12) | 0.06 |
| **End diastolic velocity** |  | |  | |  |  |
| Mean ± SD. | 3.8 ± 4 | | 5.1 ± 2.9 | | 1.15 (1.004 – 1.31) | 0.04^*^ |
| **Resistance index** |  | |  | |  |  |
| Mean ± SD. | 0.8 ± 0.1 | | 0.75 ± 0.1 | | 0.63 (0.46 – 0.88) | 0.007^*^ |
| **Initial ABG** |  | |  | |  |  |
| **PH** |  | |  | |  |  |
| Mean ± SD. | 7.3 ± 0.1 | | 7.3 ± 0.1 | | 0.16 (0.002 – 14.22) | 0.43 |
| **HCO_3_** |  | |  | |  |  |
| Mean ± SD. | 20.9 ± 3.4 | | 20.7 ± 4 | | 0.988 (0.90 – 1.09) | 0.81 |
| **PCO_2_** |  | |  | |  |  |
| Mean ± SD. | 40.7 ± 10 | | 41.2 ± 9.3 | | 1.005 (0.97 – 1.05) | 0.80 |
| **Surfactant administration** |  | |  | |  |  |
| No | 35 (79.5%) | | 68 (81.9%) | | 1.0 | 0.74 |
| Yes | 9 (20.5%) | | 15 (18.1%) | | 0.86 (0.34 – 2.16) |  |
| **Inotropes (at the time of the scan)** |  | |  | |  |  |
| No | 25 (56.8%) | | 67 (80.7%) | | 1.0 | 0.005^*^ |
| Yes | 19 (43.2%) | | 16 (19.3%) | | 0.31 (0.14 – 0.71) |  |

**S-Table (4): Multivariate logistic regression analysis for variables affecting SVC flow (final model)**

|  | Multivariate | |
| --- | --- | --- |
|  | Adjusted OR (95% C.I) | Adjusted P |
| **RSS at admission** | 0.45 (0.30 – 0.68) | <0.001* |
| **LVO** | 1.02 (1.01 – 1.024) | <0.001* |
| **Initial BC** | 0.22 (0.05 – 1.04) | 0.06 |

Multivariate binary logistic regression analysis was performed to determine factors affecting SVC flow. Backward stepwise regression was buit, with variables included in the base model if they were statistically significant (P < 0.05) in the univariate analysis. Collinearity was found between (NCPAP and conventional ventilation), (RSS at admission, RR and mean airway pressure), (CRT and PP) and (ACA RI and ACA end diastolic velocity).

Variables in the base model were as follows: gestational age, resuscitation data, RSS at admission, respiratory support (NCPAP), and inotropes at the time of scan, initial BC, CRT, LVO, RVO and ACA RI.

**S-Table (5): Univariate logistic regression analysis for normal and high ACA RI**

|  | | **ACARI** | | **Univariate** | |
| --- | --- | --- | --- | --- | --- |
|  |  | **Normal**  **(≤0.85)** | **High**  **(>0.85)** | **OR (95%C.I)** | **P value** |
| **Sex** | Male | 44 (44.9%) | 17 (58.6%) | 1.74 (0.75 – 4.03) | 0.20 |
|  | Female | 54 (55.1%) | 12 (41.4%) | 1.0 |  |
| **Gestational age** | Mean ± SD. | 29.8 ± 1.6 | 29.5 ± 1.5 | 0.90 (0.69 – 1.17) | 0.42 |
| **Weight (kg)** | Mean ± SD. | 1.1 ± 0.2 | 1 ± 0.2 | 0.27 (0.04 – 1.81) | 0.18 |
| **Maternal medical history** | |  |  |  |  |
| Anemia | | 34 | 17 | 2.67 (1.14 – 6.23) | 0.02* |
| Infection | | 17 | 13 | 3.87 (1.58 – 9.52) | 0.003* |
| Diabetes | | 3 | 0 | – | 0.999 |
| Preeclampsia | | 12 | 4 | 1.15 (0.34 – 3.87) | 0.83 |
| GHT | | 6 | 0 | – | 0.999 |
| Negative for all | | 36 | 5 | 0.36 (0.13 – 1.02) | 0.06 |
| **Antenatal steroids** | Complete course | 30 (30.6%) | 6 (20.7%) | 0.60 (0.21 – 1.72) | 0.34 |
|  | Incomplete course | 23 (23.5%) | 8 (27.6%) | 1.04 (0.39 – 2.82) | 0.93 |
|  | None | 45 (45.9%) | 15 (51.7%) | 1.0 |  |
| **Resuscitation data** | Initial steps | 70 (71.4%) | 15 (51.7%) | 1.0 |  |
|  | PPV | 17 (17.3%) | 8 (27.6%) | 2.20 (0.80 – 6.02) | 0.13 |
|  | ETT | 11 (11.2%) | 6 (20.7%) | 2.55 (0.81 – 7.96) | 0.11 |
| **Mode of delivery** | NVD | 30 (30.6%) | 13 (44.8%) | 1.84 (0.79 – 4.30) | 0.16 |
|  | CS | 68 (69.4%) | 16 (55.2%) | 1.0 |  |
| **Perinatal sentinel event** | |  |  |  |  |
| Accidental HGE | | 22 | 8 | 1.32 (0.51 – 3.38) | 0.57 |
| Cord prolapse | | 3 | 0 | – | 0.999 |
| PTLP | | 39 | 16 | 1.86 (0.81 – 4.30) | 0.15 |
| PROM | | 20 | 6 | 1.02 (0.37 – 2.83) | 0.97 |
| ROM (<18) | | 12 | 1 | 0.26 (0.03 – 2.06) | 0.20 |
| Non reassuring fetal heart rate tracing | | 5 | 0 | – | 0.999 |
| Antepartum fit | | 3 | 1 | 1.13 (0.11 – 11.30) | 0.92 |
| Doppler abnormality | | 3 | 0 | – | 0.999 |
| Obstructed labor | | 3 | 0 | – | 0.999 |
| **RSS** **at admission** | Mean ± SD. | 4 ± 1.3 | 4.7 ± 1.1 | 1.59 (1.11 – 2.26) | 0.01* |
| **MAP** | Mean ± SD. | 5 ± 2.7 | 6.8 ± 3 | 1.28 (1.08 – 1.52) | 0.005* |
| **Respiratory support** | A/C | 34 (34.7%) | 16 (55.2%) | 2.32 (0.998 – 5.38) | 0.051 |
|  | HFO | 0 (0%) | 2 (6.9%) | – | 0.999 |
|  | NCPAP | 48 (49%) | 10 (34.5%) | 0.55 (0.23 – 1.30) | 0.17 |
|  | Minimal support | 16 (16.3%) | 1 (3.4%) | 0.18 (0.02 – 1.44) | 0.11 |
| **Hb (g/dl)** | Mean ± SD. | 15.7 ± 2.2 | 14.5 ± 2.9 | 0.82 (0.68 – 0.98) | 0.03* |
| **WBC (×10^3^/ul)** | Mean ± SD. | 13.2 ± 10 | 15.8 ± 11.3 | 1.02 (0.99 – 1.06) | 0.24 |
| **Platelets (×10^3^/ul)** | Mean ± SD. | 207.1 ± 69 | 211.5 ± 87.1 | 1.001 (0.995 – 1.01) | 0.77 |
| **CRP (sec.)** | Mean ± SD. | 11.6 ± 21 | 8.8 ± 14.5 | 0.99 (0.97 – 1.02) | 0.52 |
| **PT (sec)** | Mean ± SD. | 17.9 ± 7.4 | 19 ± 8.3 | 1.02 (0.97 – 1.07) | 0.49 |
| **PTT (sec)** | Mean ± SD. | 63.5 ± 21.7 | 68.4 ± 21.6 | 1.01 (0.99 – 1.03) | 0.29 |
| **Initial BC** | Negative | 88 (89.8%) | 28 (96.6%) | 1.0 | 0.28 |
|  | Positive | 10 (10.2%) | 1 (3.4%) | 0.31 (0.04 – 2.56) |  |
| **HR (beat/min)** | Mean ± SD. | 150.8 ± 20.4 | 146.6 ± 16.6 | 0.99 (0.97 – 1.01) | 0.32 |
| **RR (breath/min)** | Mean ± SD. | 56.4 ± 11.5 | 59.3 ± 10.9 | 1.02 (0.99 – 1.06) | 0.23 |
| **CRT (sec)** | Mean ± SD. | 2.6 ± 0.8 | 3.1 ± 0.8 | 2.16 (1.29 – 3.61) | 0.003* |
| **PP** | Felt well | 85 (86.7%) | 22 (75.9%) | 1.0 | 0.16 |
|  | Weak | 13 (13.3%) | 7 (24.1%) | 2.08 (0.74 – 5.84) |  |
| **SBP (mmHg)** | Mean ± SD. | 54.1 ± 10.4 | 51.6 ± 8.8 | 0.98 (0.93 – 1.02) | 0.25 |
| **DBP (mmHg)** | Mean ± SD. | 26.6 ± 7.7 | 23.9 ± 7.1 | 0.95 (0.89 – 1.01) | 0.10 |
| **MABP (mmHg)** | Mean ± SD. | 36.1 ± 9 | 32.5 ± 8.5 | 0.95 (0.91 – 1.002) | 0.06 |
| **SVC flow (ml/kg/min)** | Low (<41) | 29 (29.6%) | 15 (51.7%) | 2.55 (1.09 – 5.95) | 0.03* |
|  | Normal (≥41) | 69 (70.4%) | 14 (48.3%) | 1.0 |  |
|  | Mean ± SD. | 59.2 ± 31.4 | 49.5 ± 31.6 | 0.99 (0.97 – 1.004) | 0.15 |
| **LVO (ml/kg/min)** | Low (<150) | 27 (27.6%) | 10 (34.5%) | 1.38 (0.57 – 3.35) | 0.47 |
|  | Normal (≥150) | 71 (72.4%) | 19 (65.5%) | 1.0 |  |
|  | Mean ± SD. | 197.9 ± 76.2 | 181.5 ± 75.8 | 0.997 (0.99 – 1.003) | 0.31 |
| **RVO (ml/kg/min)** | Low (<150) | 3 (3.1%) | 6 (20.7%) | 8.26 (1.92 – 35.53) | 0.005* |
|  | Normal (≥150) | 95 (96.9%) | 23 (79.3%) | 1.0 |  |
|  | Mean ± SD. | 308.1 ± 118 | 272.9 ± 124.2 | 0.997 (0.99 – 1.001) | 0.17 |
| **LA/AO ratio** | Mean ± SD. | 1.1 ± 0.3 | 1.2 ± 0.3 | 2.77 (0.67 – 11.42) | 0.16 |
| **PDA/weight (mm/kg)** | Mean ± SD. | 1 ± 0.9 | 1.8 ± 0.9 | 2.37 (1.47 – 3.83) | <0.001* |
| **PFO Size (mm)** | Mean ± SD. | 1.5 ± 0.7 | 1.3 ± 0.7 | 0.68 (0.38 – 1.22) | 0.20 |
| **Initial ABG** |  |  |  |  |  |
| **PH** | Mean ± SD. | 7.3 ± 0.1 | 7.3 ± 0.1 | 0.13 (0.001 – 19.03) | 0.42 |
| **HCO3** | Mean ± SD. | 20.9 ± 3.6 | 20.1 ± 4.4 | 0.94 (0.85 – 1.05) | 0.28 |
| **PCO2** | Mean ± SD. | 40.8 ± 9.3 | 41.8 ± 10.2 | 1.01 (0.97 – 1.06) | 0.64 |
| **Surfactant administration** | No | 80 (81.6%) | 23 (79.3%) | 1.0 | 0.78 |
|  | Yes | 18 (18.4%) | 6 (20.7%) | 1.16 (0.41 – 3.26) |  |
| **Inotropes at time of scan** | No | 72 (73.5%) | 20 (69%) | 1.0 | 0.63 |
|  | Yes | 26 (26.5%) | 9 (31%) | 1.25 (0.50 – 3.08) |  |

**S-Table (6): Multivariate logistic regression analysis for variables affecting ACA RI (final model)**

|  | Multivariate | |
| --- | --- | --- |
|  | Adjusted OR (95% C.I) | Adjusted P |
| **Maternal Anemia** | 2.98 (1.05 – 8.46) | 0.04* |
| **Maternal infection** | 2.57 (0.88 – 7.48) | 0.08 |
| **Hb (g/dl)** | 0.84 (0.69 – 1.03) | 0.10 |
| **CRT (sec)** | 2.03 (1.05 – 3.95) | 0.04* |
| **RVO (low)** | 5.86 (0.99 – 34.58) | 0.051 |
| **PDA/weight (mm/kg)** | 2.51 (1.42 – 4.42) | 0.002* |

Multivariate binary logistic regression analysis was performed to determine factors affecting ACA RI. Backward stepwise regression was done, with variables included in the base model if they were statistically significant (P < 0.05) in the univariate analysis. Collinearity was found between (RSS at admission and mean airway pressure).

Variables in the base model were maternal anemia, maternal infection, Hb, CRT, SVC flow, RVO, PDA/weight and MAP.


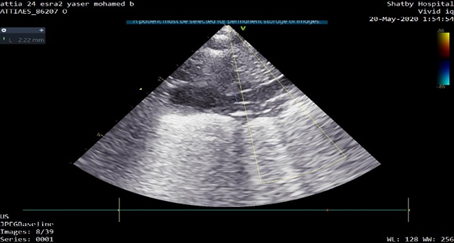

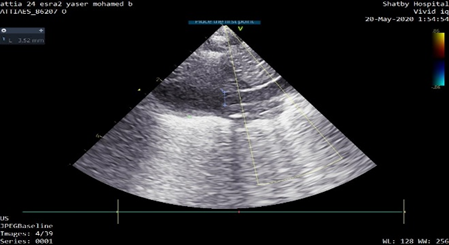


**S-Figure (2): The superior vena cava at its inlet to right atrium in the parasternal view and minimum and maximum diameters were measured.**

**
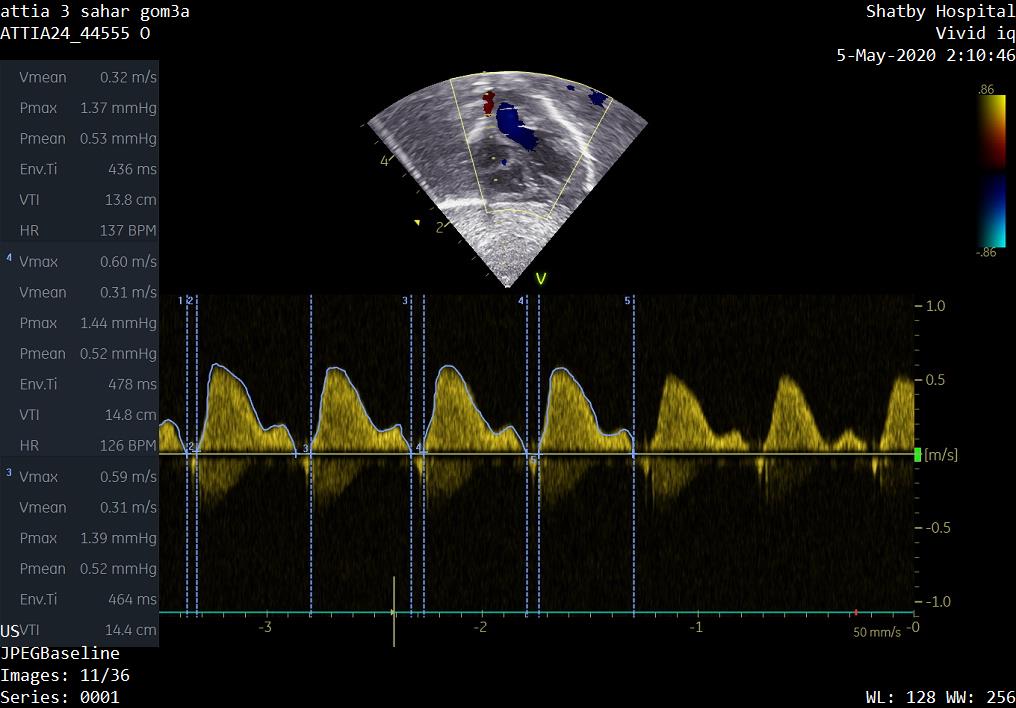
**

**S-Figure (3): Pulsed wave Doppler study of superior vena cava VTI in the subcostal view.**

**
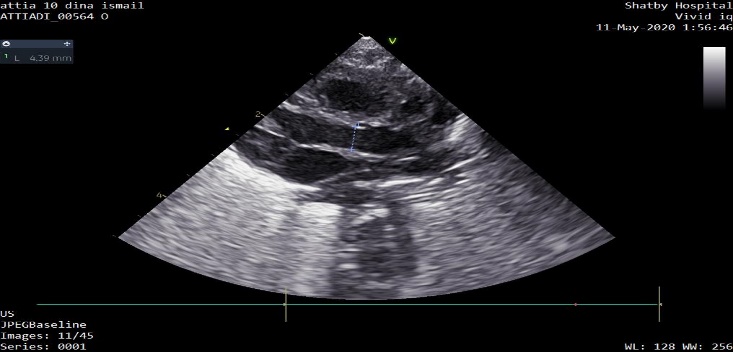
**

**S-Figure (4): Aortic root diameter assessed from the parasternal long-axis view at the valve hinge points at end systole**

**
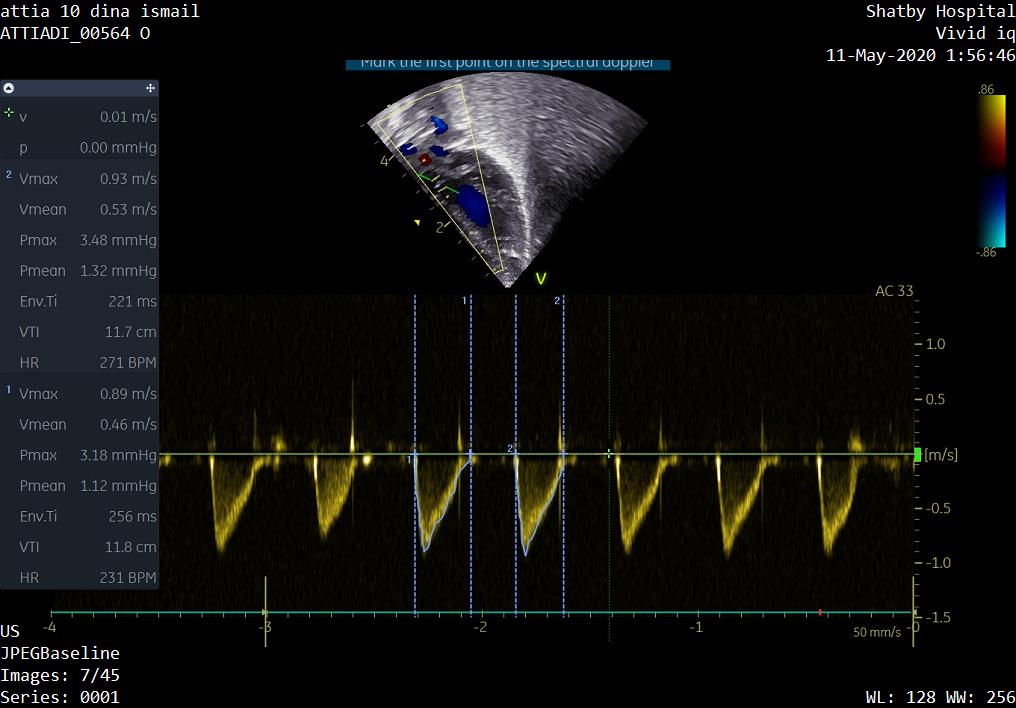
**

**S-Figure (5): Velocity time integral (VTI) was measured from an optimized apical five chamber view by placing the pulsed-wave Doppler gate at the level of the aortic valve**

**
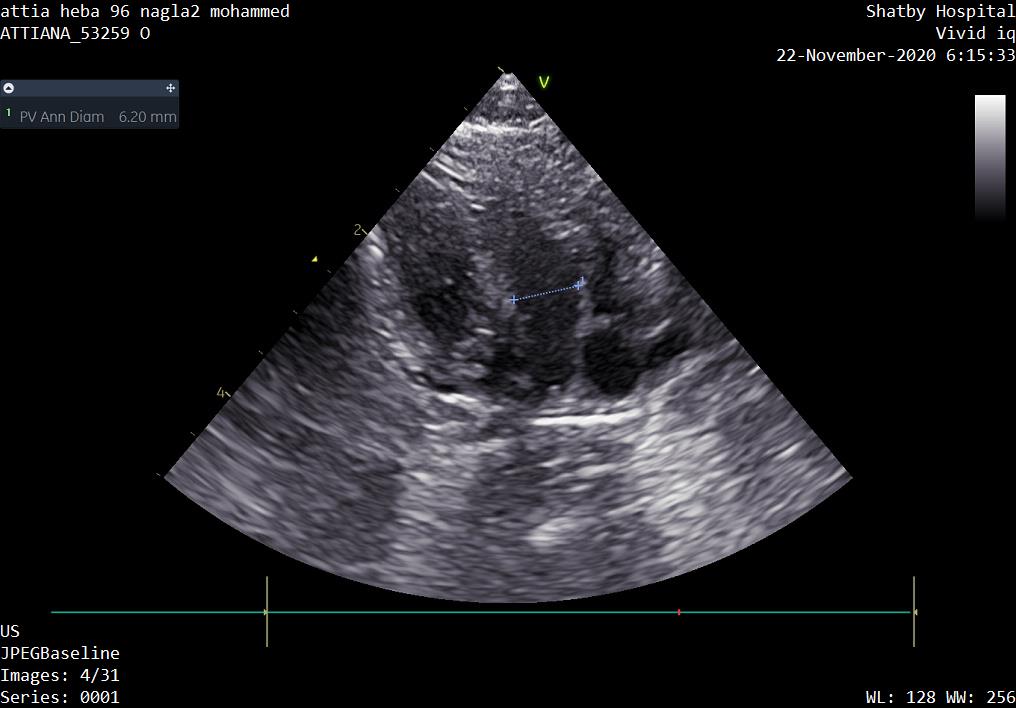
**

**S-Figure (6): The pulmonary artery diameter was measured in the end systole from an oblique long-axis parasternal view at the insertion of the pulmonary valve leaflet just before the valve closes**

**
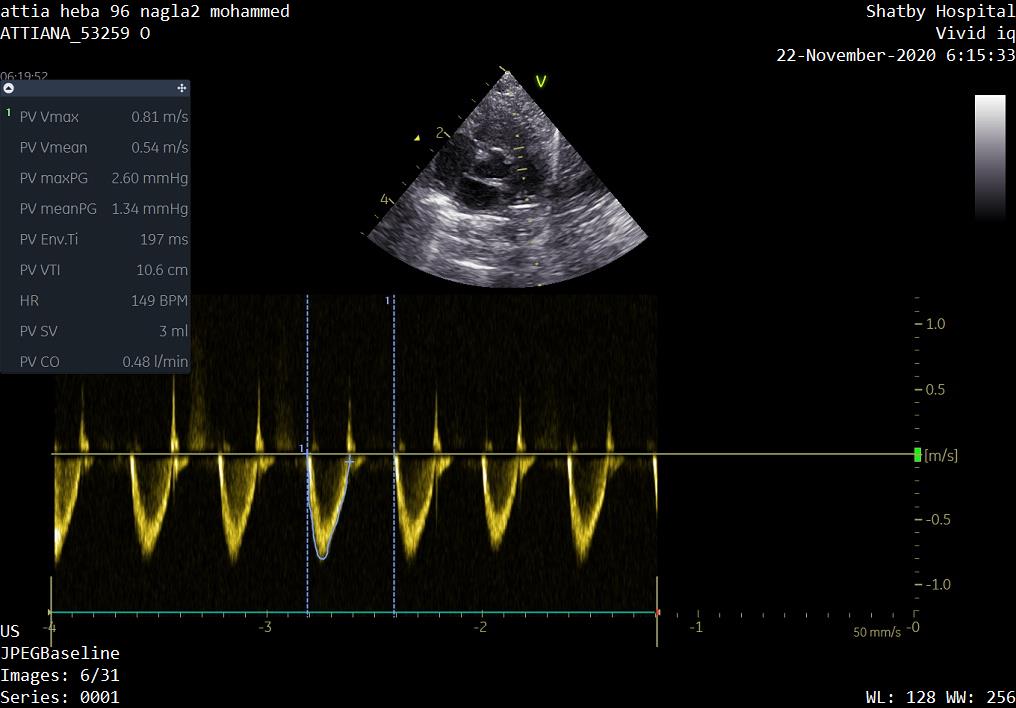
**

**S-Figure (7): The right ventricular VTI was obtained from Doppler interrogation of the main pulmonary artery in parasternal short-axis view.**

**
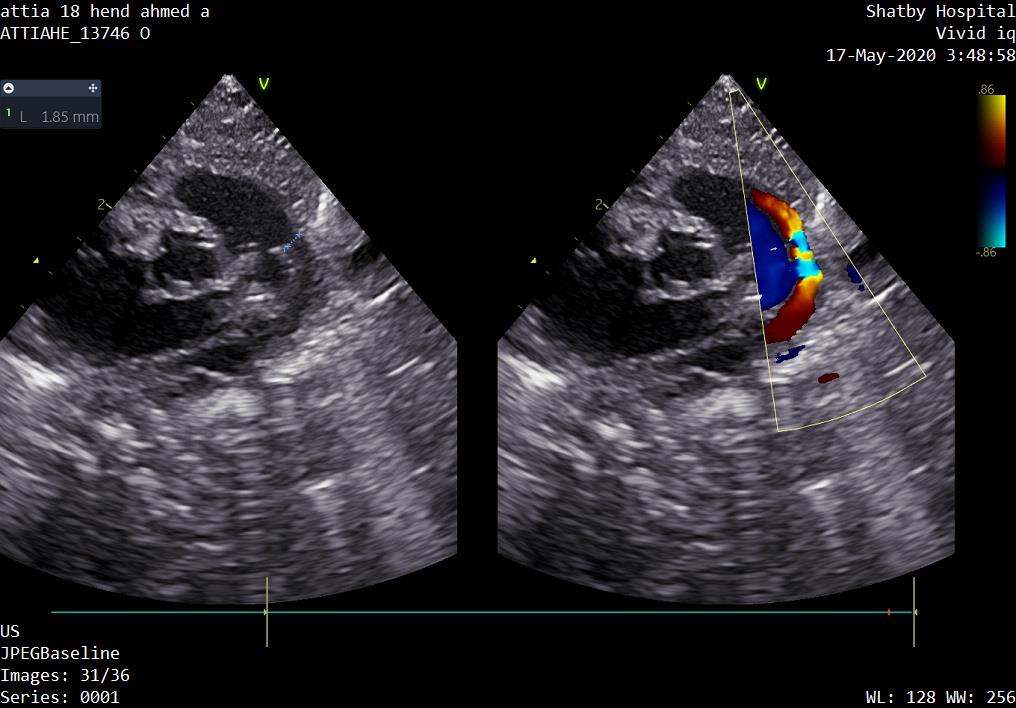
**

**S-Figure (8): Ductus arteriosus assessment from the ductal view.**

**
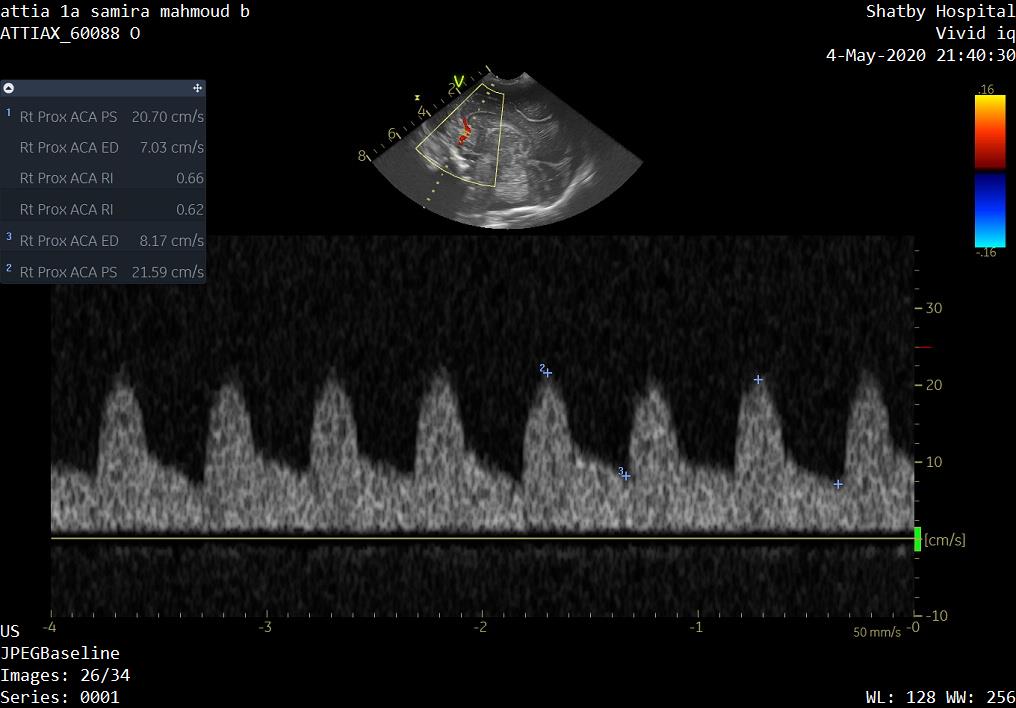
**

**S-Figure (9): Anterior cerebral artery flow velocity.**


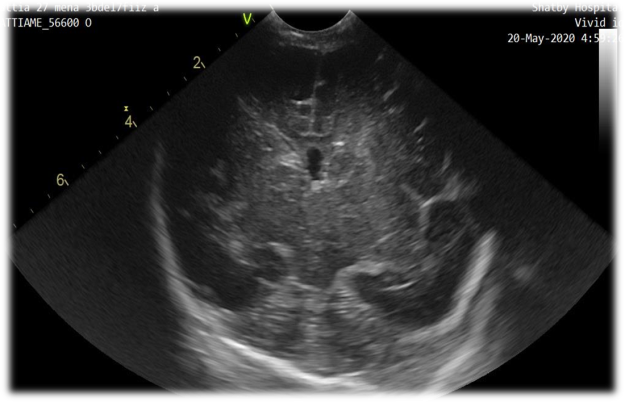

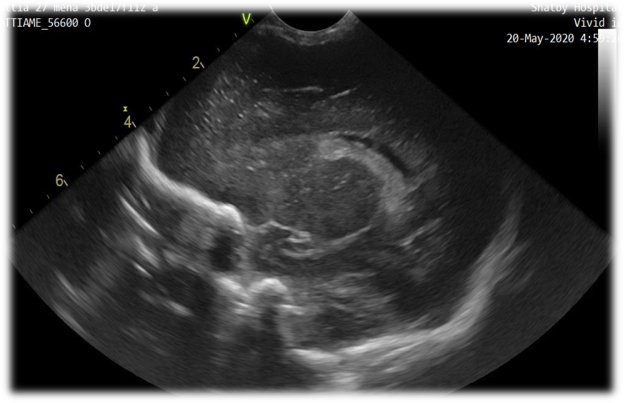


Figure10 Grade-I IVH, bleeding confined to germinal matrix


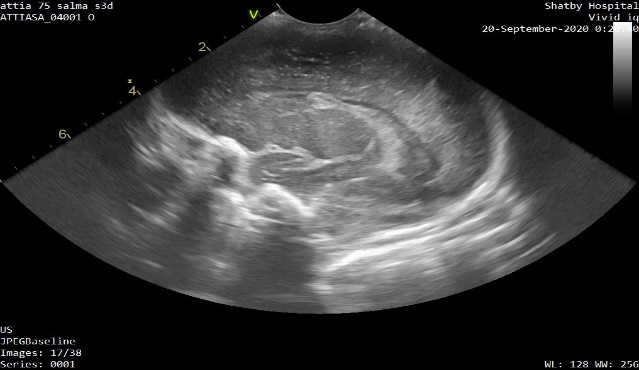


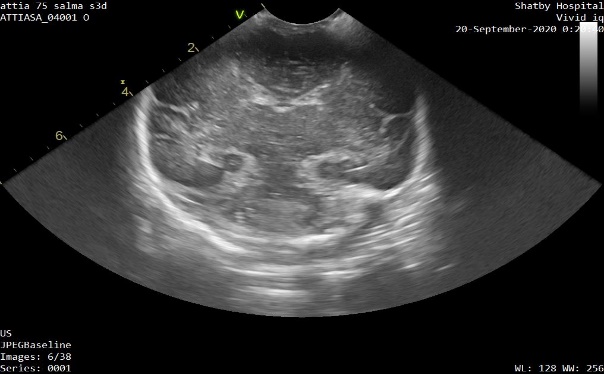


Figure11 Grade-II IVH, bleeding involves <50% of ventricle or no ventricular dilatation.


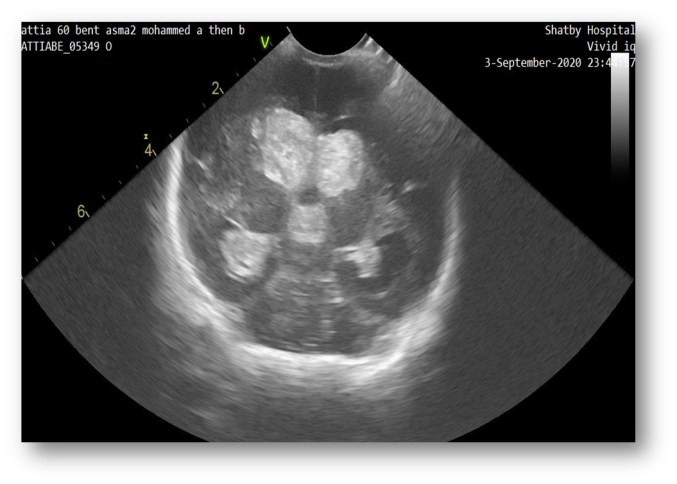

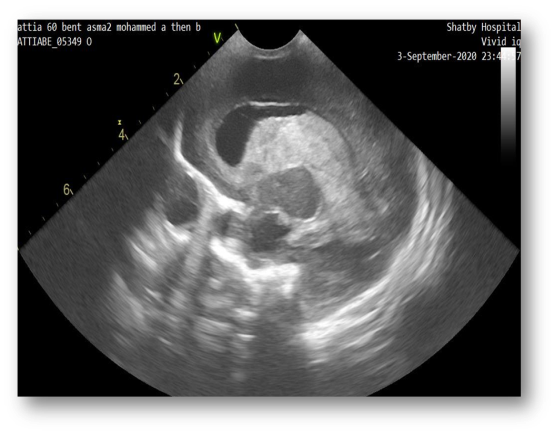


S-Figure12 Grade-III IVH, bleeding involves >50% of ventricle or ventricular dilatation


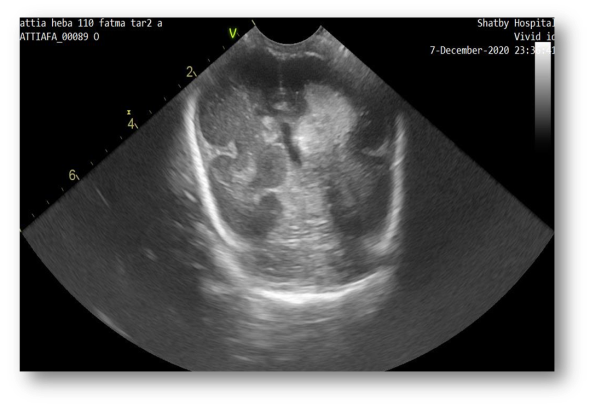

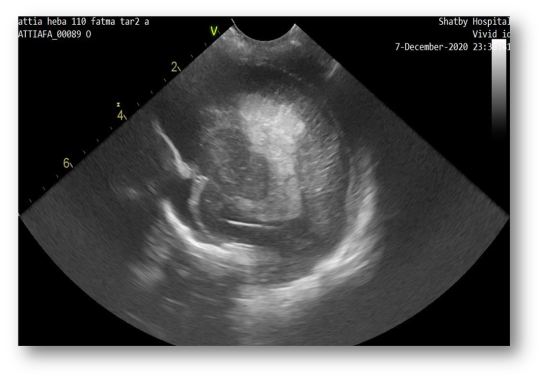


S-Figure13 Grade-IV IVH, periventricular hemorrhagic infarction, on ipsilateral side of IVH


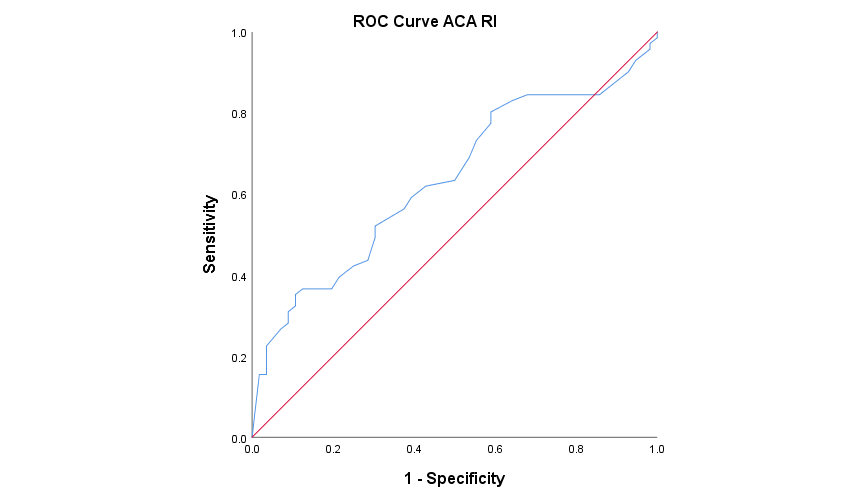
A
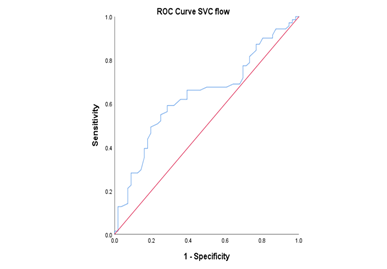
 B

S-Figure 14 **A-ROC curves for ACA-RI to predict IVH development in premature infants B- ROC curves for SVCF to predict IVH development in premature infants**

**S-Figure (15): Correlation between SVC flow and LVO with r = 0.427, p = <0.001(A) and Correlation between SVC flow and RVO,r = 0.189, p = 0.033(B)**


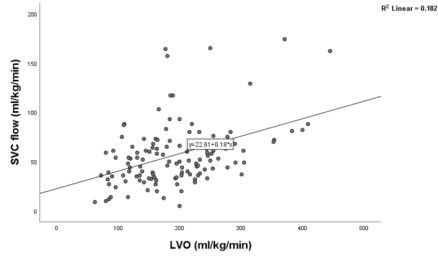


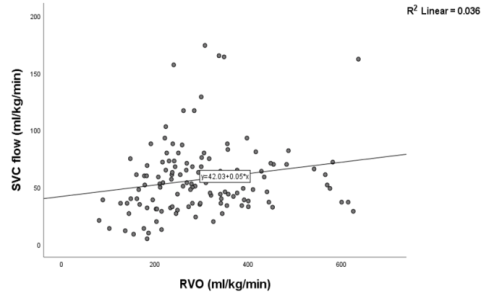
 B-


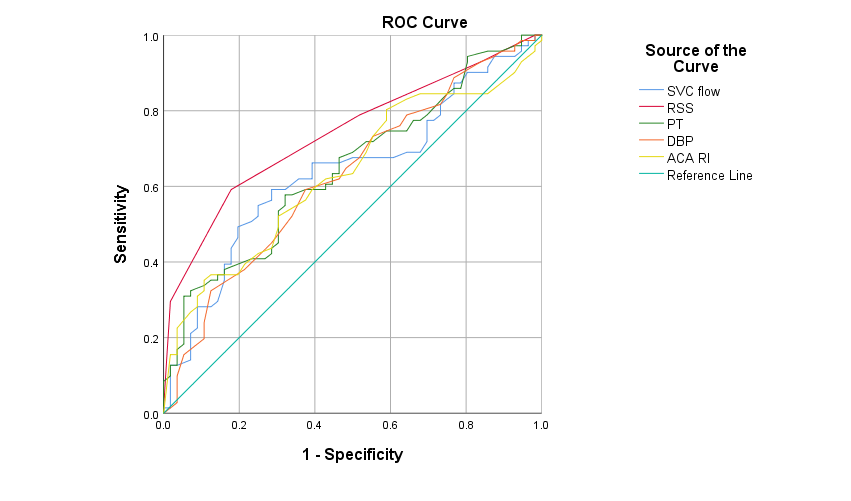


**S-Figure(16): ROC curve for the predicted probabilities of some clinical and imaging variables**

|  | **P** | **AUC** | **95% C.I** |
| --- | --- | --- | --- |
| **SVC flow** | 0.008^*^ | 0.638 | 0.542 – 0.735 |
| **ACA RI** | 0.01^*^ | 0.634 | 0.538 – 0.731 |
| **RSS** | <0.001^*^ | 0.740 | 0.655 – 0.825 |
| **PT** | 0.005^*^ | 0.647 | 0.552 – 0.742 |
| **DBP** | 0.014^*^ | 0.628 | 0.530 – 0.725 |

**S-Table(7): Area under the curve (AUC) for the clinical and imaging variables**


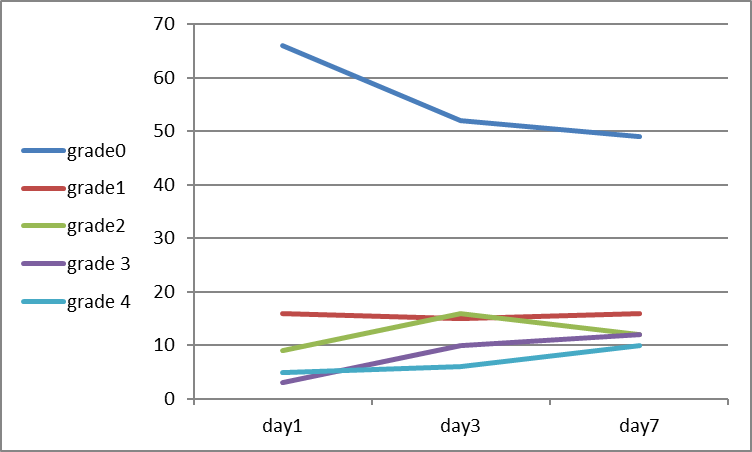


S-figure (17) A graph showing the progression of IVH incidence over time.

The incidence of IVH-I is almost stationary, while the incidence of IVH III and VI is rising over time. The incidence of IVH II is rising to D3 and then declining to D7 as some cases of IVHII progressed to grade III or VI, or died.
